# Supplementary material for: Control of the neuroprotective Lipocalin Apolipoprotein D expression by alternative promoter regions and differentially expressed mRNA 5’ UTR variants
Source: PLoS One. 2020 Jun 19;15(6):e0234857. doi: 10.1371/journal.pone.0234857 (PMC7304576; doi:10.1371/journal.pone.0234857)
Supplement: S1 Raw images — (PDF) [file pone.0234857.s001.pdf]

Raw images of Figure 3

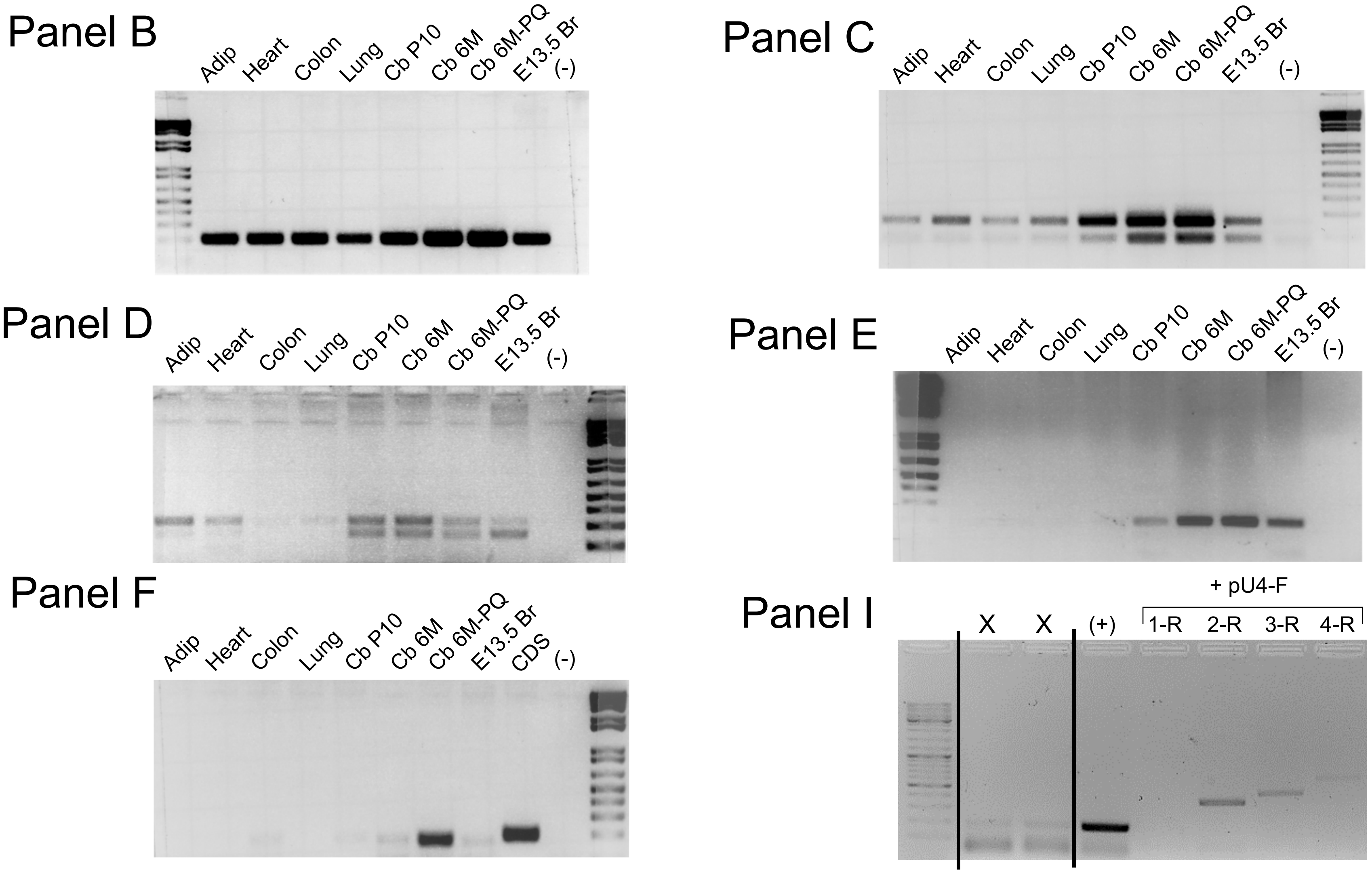

S1_raw_images

PCR amplifications were separated electrophoretically in agarose gels labeled with ethidium bromide. Bands of Panels B-F were visualized with a UV light box, pictures taken with camera attached to a thermal printer, and images digitized with a calibrated scanner. Image of Panel I was taken and digitized with a GelDoc XR system (BioRad). Relevant controls and control samples appear on the same gel as the experimental samples in every panel. Lanes marked with X in Panel I were technical control amplifications for a different experiment, and thus not included in the final figure 3I. The faint band below lane (+) in Panel I represents primer dimers of the amplification.
