# Supplementary material for: Control of the neuroprotective Lipocalin Apolipoprotein D expression by alternative promoter regions and differentially expressed mRNA 5’ UTR variants
Source: PLoS One. 2020 Jun 19;15(6):e0234857. doi: 10.1371/journal.pone.0234857 (PMC7304576; doi:10.1371/journal.pone.0234857)
Supplement: Attachment — Submitted filename: Response to reviewers PONE-D-19-34292.docx [file pone.0234857.s002.docx]

**Reviewer 1.**

We are glad the Reviewer considers our *in silico* and experimental data and findings interesting. The Reviewer raises some general considerations on the completion of our study, which we are addressing below.

*1) “The computational methodologies used by the authors are not novel and are similar to those used in the author’s previous publication…”*

The bioinformatics analysis we have performed is not “novel” (methodologically speaking, as we have not developed any new algorithm to study the ApoD upstream gene regions), but we fail to see this kind of “novelty” as a requisite in the publication criteria of *PLoS ONE*. In addition, we have used different “computational methodologies” from those used to study the Lipocalin family in our previous publication to study ApoD promoter and to design experimental molecular biology tools.

*2) “…only the in vitro assays represent novel data that isn’t readily available using online tools”*

No wonder online tools can be “readily available” for everyone. However, only if we use them to uncover interesting predictions that can be tested experimentally, they do answer pertinent questions and contribute to Science advancement. This is the case for the interesting role of upstream UTRs in Lipocalins, and the reviewers judging those in fully silico data of our previous publication raised no concern. We believe this also applies to data included in the current manuscript: ApoD mRNA has been studied for a long time as if it were a single molecular species, without noticing that an alternative promoter and diverse 5’UTR versions could condition its expression. In this work we uncover predictions and test them experimentally, which is *per se* novel.

*3) “…the authors only probed embryonic whole brain and postnatal cerebellum of mice.”*

We think the reviewer misread the information mentioned in the Methods section and shown in Fig. 3, where we describe that our PCR experiments were also performed in cerebella of adults (6 months old) mice, both control and subjected to experimental oxidative stress.

*4) “Why is the cerebellum preferentially responsive to oxidative stress?*

It was stated in line 277 of our manuscript, “Cerebellum was selected because of its reported expression of ApoD and its sensitivity to PQ [19]”. We have added more information in the introduction section (lines 71-73) to help the reader better understand this choice from the beginning.

*5) “Why did the authors choose to examine their mechanism in cultured astrocytes versus other cell types in the brain…?”*

We selected astrocyte cultures because it is one of the cell types expressing ApoD, where we have in depth knowledge about the role of ApoD in oxidative stress-dependent autocrine and paracrine protection. We explain in our manuscript (lines 435-438), that astrocytes “…show a low vesicular ApoD expression by immunolabeling under control condition, which increases when challenged with PQ (Figure 7A). This expression regulation is similar to that found in primary mouse astrocytes [14,54] and in the mouse brain upon similar oxidative stress conditions [13,18].”

*6) Statistical analysis.*

In the revised version of the manuscript, we have included a more detailed explanation of the statistical tests used in our experiments.

In a more general sense, the Reviewer poses many interesting scientific questions not addressed in this work and concludes that much work must be done before we can publish it.

Although we agree that a more detailed study could reveal more mechanistic insights and details into ApoD function regulation, we would like the Reviewer to view our data from a different perspective.

Our laboratory works with model organisms (Drosophila and mouse) on the physiology of ApoD, but is not currently funded to study in depth gene transcriptional regulation or UTR secondary structures. However, after our last publication we decided to explore ApoD 5’ UTR regions as a side project to our main grant objectives. We were able to demonstrate with standard molecular biology techniques that the predicted alternative 5’ UTRs and a novel promoter do in fact exist, and could underlie differential expression in mouse tissues and in response to oxidative stress challenges. An interesting finding acknowledged by the Reviewer.

We agree that (as always in Science) more work can be done, and would actually love to answer all questions related to this interesting topic raised by the Reviewer. However, we are also aware that our current situation prevents to keep developing that project. That is why we believe it is important to disseminate our results to the scientific community interested in transcriptional regulation, instead of hiding them in our desks waiting for a major shift in our lab goals and funding opportunities. We sincerely predict that this work would spawn future research in the field of gene expression regulation.

**Reviewer 2.**

We are glad the Reviewer considers our findings interesting, and want to thank his/her careful and useful review. We address the Reviewer’s concerns below and in the revised version of the manuscript.

*1) Statistical analysis description.*

A detailed explanation of the statistical tests used in our experiments are included in the revised version of the manuscript.

*2) References missing in the general description of ApoD function in the brain.*

Thanks for the suggestion. We have included the suggested references in the revised manuscript.

*3) Figure 2 data and design.*

Thanks for the idea. We have reorganized and expand the figure panels and legends according to the Reviewer’s suggestions.

*4) Data and methods related to the differential expression of each 5’UTR variant.*

We have revised and rewritten this section taking into account the Reviewer’s concerns. Below are our answers to these questions.

*- The primer combination used for each panel of Fig. 3 should be described.*

We have included the primer combination used in each panel of the figure. Thanks for the suggestion.

*- The difference between Fig. 3C and 3D must be explained. Were different primers used?*

Yes, those amplifications were carried out with different sets of primers. We had to use primers pU1F or pU2F to be able to separate variant A from C expression (several variants showed overlapping size). Variants B, D and E were easily identified with the primer pairs cited in the figure.

*- Table 1 should be revised.*

We have done so, and the reviewer is right about primer pU1-F and its use in RT-PCR. We have checked and corrected the experimental use of all primers listed in the revised table.

*- RNA individual samples vs. Sample pools:*

The Reviewer is right that two options can be used to account for biological and technical variabilities in experimental design. Biological variability can be evaluated by using individual samples for PCR amplifications, and technical variability would be evaluated by replicating amplifications. An alternative strategy could be to homogeneously mix equimolar amounts of RNA extracted from each individual tissue sample (obviously checking for RNA purity and integrity), thus rendering each tissue in a single pooled sample. Technical variability can be subsequently tested by experiment replicas.

We believe this approach fulfills an adequate assessment of both experimental variabilities present in biological research, and adds an economical aspect to consider (both in personal and reagent lab expenditure) for a scientist. In fact, we have been using this strategy in previous publications from our lab (Ganfornina et al 2005; Bajo et al., 2011; Sanchez et al., 2015) reporting expression data from microarray experiments, where resources and reagents costs are an issue to consider.

Moreover, the sample pooling strategy allows us to show 8 RTs (8 lanes) per PCR shown in figure 3B-F, instead of 42 RTs (and corresponding lanes) from individual sample experiments.

*- Quantification bias by contaminating residual DNA or degradation sub-products*

RNA purity and integrity were monitored by 260/230 and 260/280 ratios measured with a spectrophotometer, and by agarose electrophoresis. Putatively contaminating DNA was avoided by DNAse treatment, and confirmed by RT(-) amplifications. These details have been added to the revised Methods section.

*- Why the authors limit their investigation in brain to the cerebellum?*

As explained in the manuscript (line 277) the cerebellum was selected because of its reported expression of ApoD and its sensitivity to PQ [18]. We have added more information in the introduction section to help the reader understand this choice (lines 71-73). We agree that studying the role of 5’ UTR regulation of ApoD in neurodegenerative conditions will be very interesting, but we chose to test for the relevance of 5’ UTR variants in an experimental oxidative stress model well studied and reliably reproduced in our lab.

*- Differential expression between tissues and conditions and housekeeping gene.*

We did not include a housekeeping gene in those semiquantitative PCR experiments because we wanted to compare the expression of the 5’ UTR variants with that of ApoD CDS (shown in Fig. 3B), which in this case acts as the reference. We agree that when we refer to “differential expression of each 5’ UTR variant” is not referred to a housekeeping gene, and therefore we have rephrased the paragraph in the manuscript stating the comparison with ApoD CDS in each tissue/condition.

In quantitative real-time PCR (Fig. 3G,H), though, we used Rpl18 as a housekeeping gene.

*- Quantification of the increase in ApoD levels in IMA2.1 mouse astrocyte cells in control compared to OS conditions.*

We have performed the experiments suggested by the Reviewer, measuring ApoD expression by fluorescent immunocytochemistry and confirming the PQ-dependent overexpression. The new data was added as a new panel to Figure 7 (panel B).

*- To include full promoter region in the luciferase assays.*

We agree with the reviewer that this should be something to pursue. However, we had trouble cloning the whole DNA piece into our vectors, and proceeded to study them as separate entities.

*- Further discussion on the potential roles of the ApoD variants in control and in disease conditions.*

Although it is tempting to discuss the role of ApoD variants in human diseases, we feel our findings should be considered with caution, given their study in rodents. Thus, we have included a short statement in the revised version of the manuscript prompting the ApoD community to check the putative existence of 5’ UTR variation for the human protein, and its role in human diseases.
